# Effects of pentosan polysulfate sodium on joint structure and function out to six months in naturally-occurring canine osteoarthritis

**DOI:** 10.1371/journal.pone.0342409

**Published:** 2026-02-10

**Authors:** Catherine J. M. Stapledon, Ravi Krishnan, Rachel A. Peat, Christian Reiter, Marjorie E. Milne, Stewart D. Ryan, Sebastien H. Bauquier, Thierry Beths

**Affiliations:** 1 Paradigm Biopharmaceuticals Ltd., Melbourne, Victoria, Australia; 2 Melbourne Veterinary School, Faculty of Sciences, The University of Melbourne, Werribee, Victoria, Australia; University of Life Sciences in Lublin, POLAND

## Abstract

**Objective:**

To evaluate the durability of effect and disease modification potential of a six-week course of pentosan polysulfate sodium (PPS) therapy out to 26 weeks (six months) in companion dogs with naturally-occurring osteoarthritis.

**Design:**

Twenty mixed-breed companion dogs were enrolled and randomized to receive either subcutaneous 3 mg/kg PPS injections (n = 14) or placebo (n = 6) once weekly for six weeks. Dogs underwent assessments for pain, functional gait analysis, MRI, and biomarker analysis at baseline and selected timepoints.

**Results:**

PPS treatment was well tolerated throughout the study. At baseline, the PPS-treated group had higher pain with Helsinki Chronic Pain Index (HCPI) scores of 15.14 compared to 8.83 in placebo. PPS-treated dogs experienced sustained HCPI reductions compared to placebo at 26 weeks after adjusting for differences in baseline pain. The PPS-treated group experienced normalization of gait symmetry up to week 26, indicating a reduction in lameness and an improvement in overall function. Total cartilage volume increased at weeks 8 and 26 from baseline in the PPS-treated group compared to placebo, and OA disease progression biomarker changes (CTX-I, HA, and TIMP-1) were consistent with slowed cartilage degradation at weeks 8 and 26.

**Conclusions:**

PPS-treated dogs experienced improvements in pain, joint function, and cartilage volume compared to placebo, supported by changes in biomarkers at weeks 8 and 26. The 26-week timepoint in this translational canine model provides insights into the potential disease-modifying mechanisms and durability of PPS in long-term treatment outcomes in humans with OA.

## Introduction

Current osteoarthritis (OA) therapies such as paracetamol, opioids, and non-steroidal anti-inflammatory drugs (NSAIDs) focus on symptom management but are unable to slow or stop the underlying joint deterioration [1]. Patient dissatisfaction with current OA treatments highlights the unmet medical need for new therapies that effectively impede OA progression and improve symptoms [2]. Pentosan polysulfate sodium (PPS) is a highly sulfated semi-synthetic polysaccharide derived from beech trees. PPS has been used to treat interstitial cystitis in humans for nearly thirty years [3] and is now being developed as a potential treatment for human OA [4–6]. PPS has multiple mechanisms of action targeting inflammation, bone and cartilage degradation, and pain. Specifically, PPS has been shown to inhibit cartilage-degrading enzymes, such as a disintegrin and metalloproteinase with thrombospondin motifs (ADAMTS) −4 and −5, as well as matrix metalloproteinases (MMPs);[7] it inhibits transcription factor nuclear factor kappa-B (NF-κB) and subsequently reduces levels of pro-inflammatory cytokines, interleukin-1 beta (IL-1β) and tumor necrosis factor-alpha (TNF-α) [8–10]; while normalizing the pain mediator beta nerve growth factor (βNGF) [11].

As OA is a disease of the whole joint, PPS has the potential to improve disease outcomes, and has already been developed and approved for the veterinary treatment of OA symptoms in dogs and horses by intramuscular injection (Cartrophen Vet®, multiple jurisdictions since the 1980s; Zycosan®, USA 2022) [12]. Notwithstanding the multiple proposed mechanisms of action and its wide utilization in veterinary and human medicine, there are insufficient data supporting PPS as a disease-modifying OA drug (DMOAD). Several studies have described the therapeutic effects of intramuscular and subcutaneous PPS in canine models of naturally-occurring OA [13–15] and in surgically-induced canine OA models [16] evaluating safety, pain, and function. PPS was reported to reduce pain and lameness [13,14], improve cartilage quality [16], and reduce urinary cartilage degradation product levels [15]. However, these studies had short follow-up periods, thus were unable to assess longer-term objective measures of pain and function, nor specific effects such as structural changes to bone and cartilage as measured by imaging and systemic biomarkers.

OA is the most common canine orthopedic condition, with an annual prevalence of ~2.5% [17]. Canine OA is pathophysiologically similar to human OA, as the degenerative disorder occurs progressively and is influenced by similar risk factors such as age, genetic predisposition, excessive joint weight-bearing activity, joint malalignment, and/or previous traumatic musculoskeletal injury [18]. As the canine lifespan is considerably shorter than that of humans, normal developmental stages, as well as OA disease onset and manifestation, are represented over a shorter time frame. Therefore, the canine model of naturally-occurring OA can provide relevant translational data to evaluate DMOAD effects that would require much longer periods to assess in humans [18].

This study aimed to further evaluate potential DMOAD effects of injectable PPS in companion dogs with naturally-occurring OA of the stifle (femoro-tibio-patellar) and/or the elbow (humero-radio-ulnar) joints. Pain and function outcomes were assessed, together with structural changes as determined by MRI-based cartilage volume analysis. Serum molecular biomarkers associated with bone and cartilage degradation were also investigated. Short-term treatment effects were assessed at 8 weeks (2 weeks after final injection) and durability of treatment response at 26 weeks.

## Materials and methods

### Ethics approval and study design

Companion dogs presenting to the University of Melbourne Green Cross Animal Hospital facility in Werribee (formerly UVet Hospital) for signs of lameness, with or without previously diagnosed OA, were recruited via direct email or social media. The study occurred from February 2022 to December 2023 and was approved by the University of Melbourne Animal Ethics Committee (Ethics ID: 22714). Informed written consent was obtained from each dog’s owner prior to study enrollment and animal care was in accordance with institution guidelines. Inclusion criteria assessed at baseline (screening) included age (≥7 years), weight (between ≥15 and ≤35 kg), neuter status (previously desexed), general good health with normal blood hematology and biochemistry, radiographic evidence of OA (stifle or elbow) and pain/joint dysfunction (determined by Helsinki Chronic Pain Index [HCPI] and gait analysis), normal neurological exam, and a healthy body condition score between 4–7 (S1 Table) [19]. Dogs were deemed ineligible if they presented with comorbidities, abnormal blood hematology or biochemistry results, a previous history of PPS treatment, and/or treatment for OA within the last 14 days (S2 Table).

If pain was deemed “worse” anytime throughout the study, owners could administer anti-inflammatory rescue medication (2 mg/kg/day grapiprant [Elanco, Australia]). Due to known washout periods and observed clinical effects, rescue medication may have impacted study outcomes if it was administered for >5 consecutive days and took place within four weeks prior to clinical assessment timepoints [20]. To determine whether this occurred, rescue medication impact was classified depending on the administration date and duration relative to assessment dates.

### Study group randomization and treatment administration

If deemed eligible, dogs were randomized 2:1 to receive PPS (3 mg/kg, equivalent to 1.8 mg/kg human dose [HED]) or placebo (saline [sodium chloride 0.9%] Baxter Viaflex) by a veterinarian using the Microsoft Excel software ‘*rand’* function. Confounders were not controlled for. After randomization, dogs received a subcutaneous injection of either PPS or placebo once weekly for six weeks. Injections were administered using a sterile 1 mL syringe with a 22 G needle, whilst the dog was sitting or standing. An unblinded veterinarian prepared the study drug and an unblinded veterinary nurse ensured correct treatment allocation. Neither was involved in clinical evaluation. To reduce the chance of accidental unblinding, owners always waited in a consultation room while the dogs were injected in a separate preparation room.

### Quality of life questionnaire (Helsinki Chronic Pain Index)

Owners completed the HCPI assessment at baseline, and weeks 3, 6, 8, and 26. The HCPI—consisting of 11 questions—is a well-validated, reliable tool to assess treatment response in dogs with OA [21]. Responses were assigned a value between 0–4, equating to a total index score range of 0–44, with 0 indicating no pain and 44 extreme pain. Individual question scores of 0 and 1 indicated normal behavior and movement, where scores of 2, 3, and 4 indicated pain of increasing severity. Healthy dogs score between 0–11, whereas dogs with chronic pain score from 12–44 [22,23]. There was no minimum HCPI pain score for inclusion into the study. The owners were masked to the treatment until their animal was discharged from the study.

### Objective gait analysis

Objective gait analysis was performed at baseline and weeks 3, 6, 8, and 26 using the GAIT4Dog® Portable Walkway System (CIR Systems Inc., NJ, USA), a low-profile temporospatial pressure-sensing walkway that captures multiple sequential foot strikes. Dogs were walked two to three times on the 7-metre walkway by a study handler to become familiar with the walkway, handler, and leash, prior to recording. Recordings were considered valid when all four limbs fully contacted the walkway and the dog walked forward without stopping, hesitating, or overt head movements. The mean of three recordings was analyzed. All walkway system data were exported to Microsoft Excel and a weight-bearing measure—the total pressure index (TPI%)—was obtained for gait analysis. TPI% is the sum of peak pressure from each sensor activated by a paw during mat contact. In healthy dogs, forelimbs carry 60% of the dog’s total weight (thus 30% TPI for each forelimb) with the hindlimbs bearing 40% (20% for each hindlimb) [24].

The symmetry index (SI) is a measure of relative limb dysfunction, and was determined by calculating the difference between TPI% of affected and a non-affected limb as follows: [25]


SI=affected limb TPI%/non−affected limb TPI% 

### Anesthesia

Anesthesia was administered to the dogs to facilitate collection of biological samples (blood, synovial fluid, and urine) and imaging of limb joints by X-ray and MRI. Dogs were premedicated with intramuscular (IM) quadriceps injections of butorphanol (0.2 mg/kg; Randlab, Australia) and or medetomidine (0.005–0.01 mg/kg; Ilium, Troy, Australia) using a 22 G needle. Fifteen minutes post-administration, an intravenous (IV) catheter was placed in a cephalic vein (using an 18–22 G needle depending on dog size). Anesthesia was induced with IV propofol lipuro 1% (2–4 mg/kg; B Braun, Australia). An endotracheal tube was placed and connected to an anesthesia machine using a rebreathing system (Carescape B650 Monitor, GE Healthcare, Australia). Anesthesia was maintained with isoflurane (1–2%, Isoflo, Zoetis, Australia) in oxygen (1–2 L/min). Monitoring consisted of ECG, capnography (respiratory rate and carbon dioxide), pulse oximetry, non-invasive blood pressure and temperature. Hartmann’s fluid (Baxter Healthcare, Australia) was administered at a rate of 2.5–5 mL/kg/hour. Once the procedures were complete, all anesthesia was stopped, and dogs recovered on breathing oxygen. Following return of the swallowing reflex, dogs were extubated and sent to the ward where observation was conducted until they were ready to be discharged.

### Magnetic Resonance Imaging (MRI) and analysis

Dogs were anesthetized prior to MRI at weeks 1, 8, and 26. MRI was conducted on a Phillips Ingenia 3T machine (Philips Healthcare, Amsterdam, Netherlands). MRI of the stifle joint was conducted using a dS Small Extremity 16Ch coil (Invivo Corporation, FL, USA) on 12 PPS-treated and 5 placebo dogs. For MRI of the elbow joint the dS Flex S coil was used (Invivo Corporation) on 2 PPS-treated and 1 placebo dogs. The following MRI sequences were acquired: transverse plane proton-density weighted (PDW) and PDW with fat suppression, T2-weighted imaging (T2W) with fat suppression; dorsal plane T1-weighted imaging (T1W), PDW, and PDW with fat suppression; sagittal plane T1W, PDW with fat suppression, T2W with fat suppression, and 3D water-selective cartilage scan (WATSc) sequences.

Sagittal PDW and WATSc image sequences were analyzed. Sagittal slices were traced by a specialist veterinary radiologist, masked to treatment groups, using the ITK-SNAP software (free open-source software; www.itksnap.org) to quantify cartilage volume (mm³) of the femoral condyle, tibial plateau, and patella regions of the canine stifle [26]. Total cartilage volume was the sum of the three respective regions. Dogs with elbow OA were not included in cartilage volume analysis due to small numbers (n = 3) and regional differences.

### Biochemistry and serum biomarker analysis

Blood samples were obtained from all dogs under anesthesia at baseline, and study weeks 1, 8, and 26. Following serum collection and preparation, samples underwent full biochemistry and hematology analysis or were stored at −80°C until analysis. Enzyme-linked immunosorbent assay (ELISA) validation with canine antigens was performed by Nordic Bioscience (Herlev, Denmark). The following serum biomarkers were then assessed; C-terminal telopeptide I (CTX-I) [Roche Diagnostics, Basel, Switzerland], type II collagen cleavage product (C2C) [IBEX, DC, USA], neo-epitope fragment of type III collagen generated by matrix metalloproteinase-9 cleavage (C3M) [Nordic Bioscience], hyaluronic acid (HA) [Corgenix, CO, USA], released N-terminal pro-peptide of type II collagen (chPRO-C2) [Nordic Bioscience], and tissue inhibitor matrix metalloproteinase 1 (TIMP-1) [MyBioSource, CA, USA].

### Statistical analysis

No formal power calculations were performed to assess sample size in this exploratory pilot study. Data was analyzed for normality and mean, standard deviation (SD), and 95% confidence intervals (95% CI) were generated. A two-way ANOVA with Sidak’s multiple comparisons was applied to datasets comparing PPS and placebo groups. P-values are accompanied by 95% CI of the mean difference. Due to small group numbers, the Hedges’ *g* power analysis test was used to determine a true treatment effect (effect size) in the presence or absence of a statistically significant p-value. Hedges’ *g* power analysis was conducted using the following equation:


$$\begin{array}{c} g=\bar{y}\text{1}-\bar{y}\text{2}/sp \end{array}$$

Where ӯ1, ӯ2, and sp denote the sample 1 mean, the sample 2 mean, and the pooled standard deviation, respectively. Effect sizes are independent of the sample size and are statistically categorized as small (0.20–0.49), medium (0.50–0.79), or large (>0.80) [27]. Missing values due to sample dropout were registered and reported but excluded from further analyses. All statistical analyses were performed and graphs generated with GraphPad Prism version 10 software (GraphPad Software Inc., CA, USA).

Due to the baseline effect estimate (−9.65%) for the HCPI dataset, a mixed model for repeated measures (MMRM) analysis was performed generating adjusted least squares (LS) mean percentage change from baseline (%CfB) values. The MMRM included factors for treatment group (PPS, placebo), visit, treatment-by-visit interaction, baseline HCPI value, and baseline-by-visit interaction. Fixed effects of group, week, and group*week interactions were considered along with repeated measures of baseline scores.

## Results

### Cohort demographics and baseline clinical features

Following informed consent, 21 dogs met the inclusion criteria and were enrolled in the study between April 2022 and June 2023. One dog was removed from the study due to abnormal baseline biochemistry results. A total of 20 dogs completed the study (PPS n = 14; placebo n = 6), and included the following breeds: Australian Shepherd, Beagle, Blue Heeler, Boxer, Border Collie, German Shepherd, Kelpie cross, Labrador Retriever, Labradoodle, Staffordshire Bull Terrier, and Staffordshire Bull Terrier cross. Twelve dogs were castrated males, and eight were spayed females. Dog age and weight ranged between 7–13 years and 16.7–34.4 kg, respectively (Table 1).

**Table 1:** Study group characteristics at baseline.

	PPS (n = 14)	Placebo (n = 6)
Sex	Males (n = 8) Females (n = 6)	Males (n = 4) Females (n = 2)
Age, years mean ± SD (95% CI)	10.2 ± 1.9 (9.07, 11.31)	9.2 ± 2.1 (6.70, 11.41)
Body weight, kg mean ± SD (95% CI)	24.7 ± 4.27 (22.21, 27.14)	29.3 ± 3.54 (25.53, 32.70)
Breeds	11 purebred dogs and 3 crossbreed dogs	5 purebred dogs and 1 crossbreed dog
OA index joint*	Elbow (n = 7) Stifle (n = 7)	Elbow (n = 2) Stifle (n = 4)
Pain on HCPI mean ± SD (95% CI)	15.1 ± 8.18 (10.42, 19.69)	8.8 ± 2.79 (5.91, 11.76)
Body condition score mean ± SD (range)	3.4 ± 0.50 (3–4)	3.3 ± 0.51 (3–4)

*Index joint was determined upon gait assessment (TPI%). Abbreviations: CI, confidence interval; HCPI, Helsinki Chronic Pain Index; kg, kilogram; OA, Osteoarthritis; PPS, pentosan polysulfate sodium; SD, standard deviation; TPI%, total pressure index.

### Drug safety and clinical biochemistry

PPS was well tolerated with no reports of adverse events. Routine blood hematology and biochemistry conducted at baseline and at week 26 did not identify any abnormalities in markers of immune, liver, kidney, or platelet function. No unexpected adverse events in dogs treated with PPS or placebo treatment were reported by owners between timepoints (S2 Table).

### Rescue medication administration

No study dogs required pain medication at baseline. Of the dogs that did receive rescue medication, the administration timing may have affected HCPI scoring and/or gait analysis at one or more timepoints for one PPS-treated dog and two placebo dogs. No six-month timepoint assessments were impacted by rescue medication (Table 2).

**Table 2:** Administration and likely impact of rescue medication.

PPS-Treated Group			
Dog number	Timepoint	Duration (days)	Likely study impact (Y/N)
2	Week 22	5	No
6	Week 8*	10	No
7	Week 0*	5	No
9	Week 1	4	Yes
11	Week 26*	5	No
12	Week 0*	5	No
16	Week 8*	5	No
18	Week 8*	10	No
Mean (95% CI) Rescue Medication Administration (Days)		6.1 (4.11, 8.15)	
**Placebo Group**			
**Dog number**	**Timepoint**	**Duration (days)**	**Likely study impact (Y/N)**
1	Week 4	10	Yes
	Week 26*	10	No
14	Week 1*	10	Yes
21	Week 0*	5	No
	Week 8*	5	No
Mean (95% CI) Rescue Medication Administration (Days)		8.0 (4.60, 11.40)	

For each dog, rescue medication (grapiprant) was available at the request of the owner if pain symptoms worsened at any time for their dog during the study. A dose of 2 mg/kg was administered at various timepoints and periods for dogs in both the placebo and PPS-treated groups. Any rescue medication administered in the four weeks preceding an assessment was considered likely to have an impact on pain or gait assessments. If rescue medication was administered outside of these specific timeframes, it was considered to have no impact on assessments. Furthermore, rescue medication administered outside the study period had no impact on results. *Rescue medication was administered after assessments. Abbreviations: CI, confidence interval; N, no; PPS, pentosan polysulfate sodium; Y, yes.

### The Helsinki Chronic Pain Index (HCPI) questionnaire

**Fig 1:**
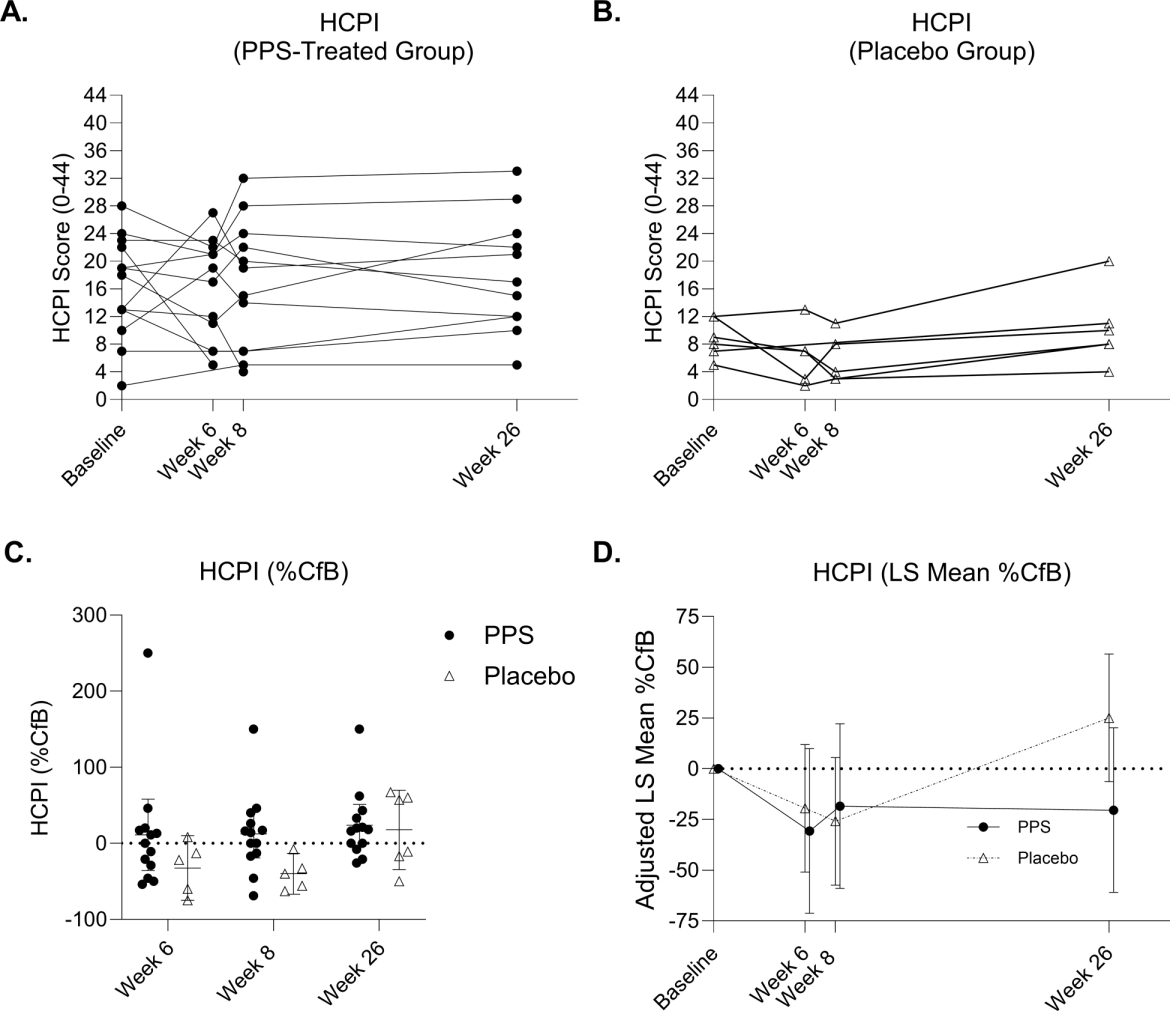
Effect of PPS treatment on dog pain following owner-completed Helsinki Chronic Pain Index (HCPI) assessment. Spaghetti plots for Helsinki Chronic Pain Index (HCPI) scores for individual animals in **(A)** PPS-treated group at baseline, week 8 and week 26 and **(B)** placebo group at baseline, week 8 and week 26. The mean HCPI value is represented as a dotted line; **(C)** Histogram with percentage change from baseline (%CfB) at week 6 versus 8 (p = 0.42) and week 8 versus 26 (*p = 0.006); **(D)** HCPI (least squares [LS] mean %CfB). Data are displayed as mean ± 95% CI and analyzed by a two-way ANOVA. with Sidak’s multiple comparisons test. LS Mean %CfB data are displayed as mean ± 95% CI. The n for each group and timepoint corresponds to available HCPI questionnaire responses.

At baseline, the PPS-treated group had higher pain levels compared to placebo with mean (95% CI) HCPI scores of 15.1 (10.42, 18.87) versus 8.8 (2.11, 11.89), p = 0.02 (95% CI: 1.124, 11.490) (Fig 1A and 1B). Fig 1C demonstrates the mean percentage change from baseline (%CfB) in HCPI over time. Due to higher baseline HCPI scores in the PPS-treated group, MMRM analysis was performed (Fig 1D). LS mean (%CfB) HCPI was reduced in both groups at week 6 (final injection), with a greater reduction in the PPS-treated group (−30.7% [−71.22, 9.90]) versus placebo (−19.5% [−50.90, 11.95]), where >5%CfB is considered the threshold for a minimal clinically important difference (MCID) [28]. LS mean (%CfB) HCPI remained below baseline at week 8 in both groups. At week 26, the PPS-treated group had sustained HCPI reduction (−20.4% [−60.94, 20.18]), whereas placebo pain increased (25.1% [−6.33, 56.52]) (Fig 1D).

### Lameness assessment (gait analysis)

PPS treatment effects on OA-associated lameness were assessed via objective gait analysis (force and symmetry). As only two placebo dogs had affected elbows (Table 1), gait analysis was limited to the poorest performing (index) stifle. Optimal TPI% percentages are 20% for each hindlimb due to relative weight distribution [28].

**Fig 2:**
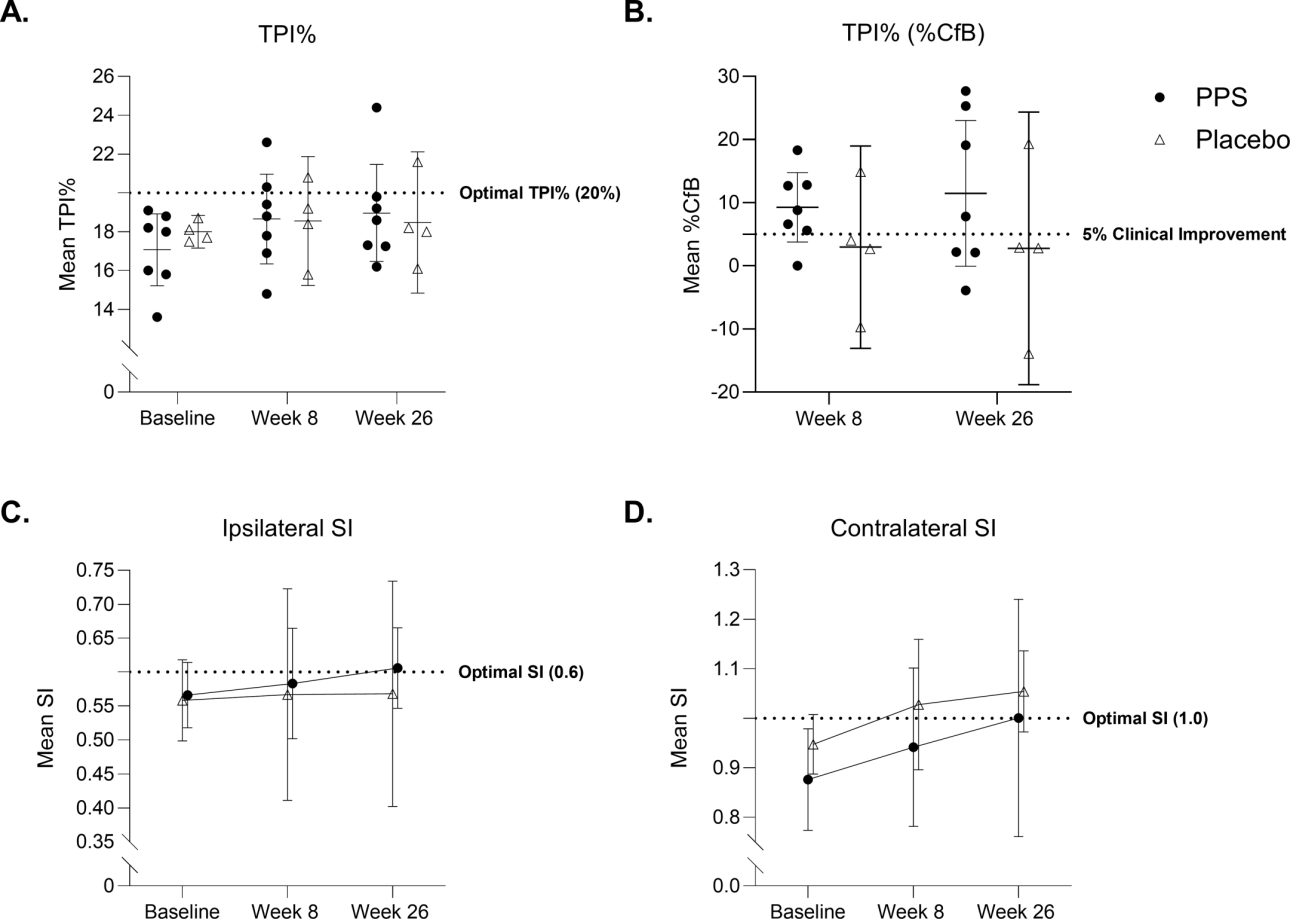
Effect of PPS treatment on index stifle osteoarthritic gait and symmetry. Total pressure index (TPI%) and symmetry index (SI) analysis. PPS is represented by filled circles and placebo by open triangles. **(A)** Index hindlimb TPI% for PPS and placebo groups. **(B)** Percentage change from baseline (%CfB) analysis of TPI%. A 5% or greater increase in the %CfB value was considered to be a clinically meaningful improvement. **(C)** Ipsilateral SI values from week 1 to week 26. **(D)** Contralateral SI values from week 1 to 26. Data are represented as mean ± 95% CI. The n for each group and timepoint corresponds to valid gait assessments.

At baseline, the PPS-treated group had a lower mean (95% CI) TPI% compared to placebo (17.0% [15.22, 18.92] versus 18.0% [17.16, 18.84]) respectively, p = 0.28 (−0.950, 2.807). A positive unidirectional shift in TPI% towards the optimal 20% following PPS treatment indicated normalization of asymmetric gait at week 8 (18.7% [16.35, 20.97]) and at week 26 (19.0% [16.47, 21.46]) [28]. Conversely, placebo worsened by shifting towards asymmetric gait at week 8 (18.6% [15.23, 21.87]), p = 0.94 (−3.397, 3.183), changing to 18.5% (14.83, 22.12), p = 0.76 (−4.081, 3.102) at week 26 (Fig 2A). At week 8 in the PPS-treated group, there was a clinically meaningful change (>5%) in TPI% percentage change from baseline (%CfB [95% CI]) of 9.3% (3.75, 14.76), which was not observed in placebo (2.8% [−13.04, 18.99]), p = 0.35 (−20.180, 7.612) [29,30]. Similar results were observed at week 26 in the PPS-treated group (11.5% [−0.08, 23.03]) versus placebo (3.0% [−18.79, 24.34]), p = 0.21 (−22.590, 5.198) (Fig 2B).

Symmetry index (SI), a derivative TPI% measure, assesses limb dysfunction. In healthy dogs, contralateral (left and right fore or hindlimbs) SI values should approach 1.0 and ipsilateral (same side hind and forelimbs) ~0.67 [28,31].

Ipsilateral SI (95% CI) at baseline deviated from the optimal value of ~0.67 in PPS-treated (SI = 0.57 [0.518, 0.614]) and placebo groups (SI = 0.56 [0.498, 0.618]), p = 0.79 (−0.071, 0.055). At weeks 8 and 26 the PPS-treated group approached the optimal ipsilateral SI (0.58 [0.502, 0.665] and 0.61 [0.547, 0.665] respectively), whereas placebo remained stable at weeks 8 and 26 (0.57 [0.411, 0.723], p = 0.79 (−0.163, 0.129) and 0.57 [0.402, 0.734], p = 0.54 (−0.192, 0.116), respectively) (Fig 2C).

Contralateral SI (95% CI) at baseline deviated from the optimal 1.0 in PPS-treated and placebo groups (SI = 0.88 [0.774, 0.979] and 0.95 [0.887, 1.008], respectively), p = 0.17 (−0.036, 0.179). At week 8, PPS-treated SI shifted towards 1.0 (from 0.88 to 0.94 [0.782, 1.101]), and continued to improve, attaining normalized gait by week 26 (1.00 [0.761, 1.240]). Whereas placebo shifted away from 1.0 (from 0.95 to 1.03 [0.896, 1.159]) at week 8, p = 0.31 (−0.091, 0.264) and shifted further towards asymmetry at week 26 (1.05 [0.973, 1.136]), p = 0.63 (−0.189, 0.296), indicating overcompensation on the contralateral side (Fig 2D).

### Quantitative MRI analysis

**Fig 3:**
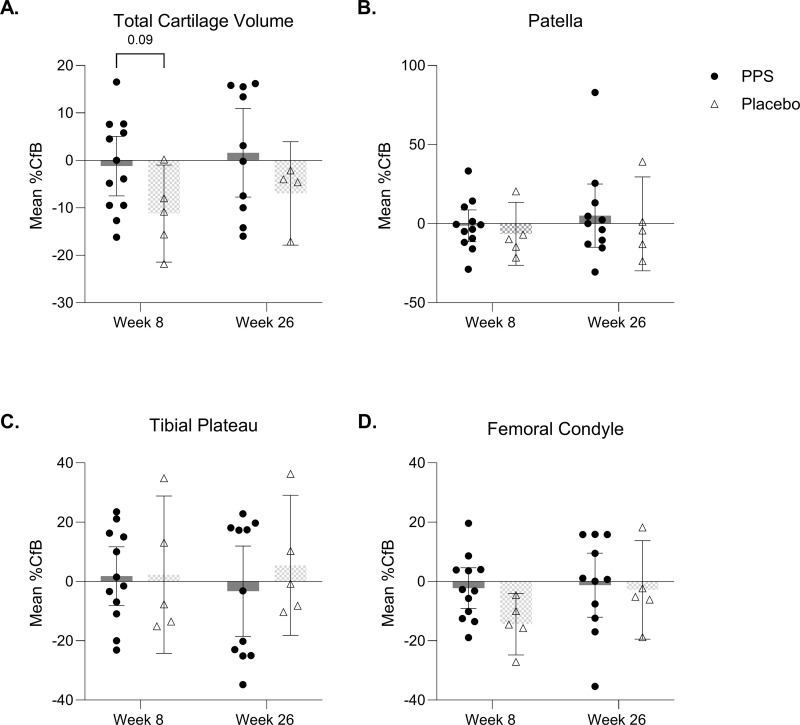
Quantitative MRI analysis of cartilage volume (mm^3^) in the canine stifle joint. Percentage change from baseline (%CfB) in cartilage volume at week 8 and week 26 for: **(A)** Total cartilage volume; **(B)** Patella; **(C)** Tibial plateau; **(D)** Femoral condyle. Data is displayed as mean ± 95% CI and analyzed by two-way ANOVA with Sidak’s multiple comparisons. The n for each group and timepoint corresponds to valid MRI measurements.

MRI sequences were acquired at baseline, weeks 8, and 26. At baseline, PPS-treated mean [95% CI] total cartilage volume was ~ 16% lower than placebo (865 mm^3^ [640.48, 1049.63] versus 1006 mm^3^ [782.37, 1230.22]) respectively, p = 0.25 (−111.9, 394.4). This difference suggests a higher baseline degree of disease in the PPS-treated group, perhaps contributing to the increased pain. At week 8, cartilage loss was lower in PPS-treated dogs than in placebo, with mean %CfB (95% CI) cartilage volume of −1.2% (−7.47, 5.05) versus −11.2% (−21.44, −0.96), respectively, p = 0.09 (−21.51, 1.523), effect size 1.06. At week 26, cartilage volume was slightly increased in the PPS-treated group with %CfB of +1.6% (7.72, 10.94) (effect size 0.73; S3 Table) versus a loss of −7.0% (−17.85, 3.95) in placebo, p = 0.13 (−21.359, 3.061) (Fig 3A).

Mean %CfB (95% CI) patella cartilage volume was reduced at week 8 in PPS-treated (−1.3% [−11.38, 8.71]) and placebo groups (−6.5% [−26.31, 13.39]), p = 0.67 (−29.80, 19.54) (Fig 3B). However, at week 26, the mean patella cartilage volume (%CfB) increased 5.1% (−14.98, 25.11) in the PPS-treated group compared to baseline, versus a slight loss of −0.1% (−29.77, 29.57) in placebo, p = 0.68 (−30.50, 19.87). Conversely, although tibial plateau cartilage volume %CfB increased slightly in PPS-treated and placebo groups at week 8 (1.8% [−8.09, 11.69] versus 2.3% [−24.20, 28.84]) respectively, p = 0.99 (−24.01, 25.05) (Fig 3C), by week 26 it further increased in placebo (5.5% [−18.17, 29.09]) and decreased in the PPS-treated group (−3.3% [−18.94, 11.95]), p = 0.70 (−16.74, 32.89).

Femoral condyle cartilage volume %CfB reduced in the PPS-treated group at weeks 8 (−2.2% [−9.12, 4.64]), and 26 (−1.2% [−12.02, 9.57]). The placebo group experienced greater femoral condyle cartilage loss of −14.4% (−24.74, −4.02), p = 0.17 (−28.39, 4.110) at week 8, and −2.8% (19.43, 13.75), p = 0.98 (−17.65, 15.21), at week 26 (Fig 3D).

### Serum biomarker analysis

**Fig 4:**
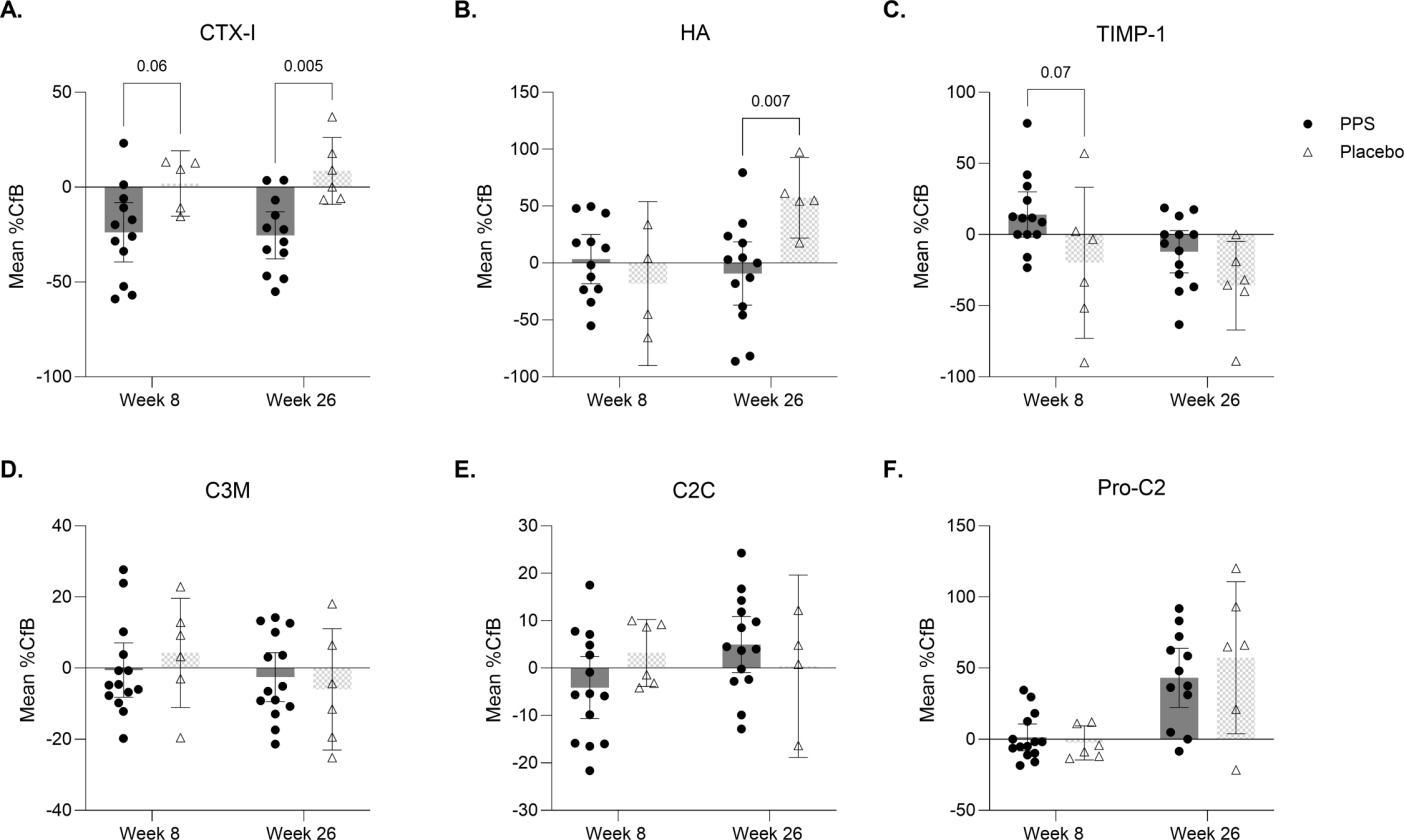
Serum biomarker ELISA analysis. Analysis of serum biomarkers of cartilage and bone degradation, as well as inflammation by enzyme-linked immunosorbent assay (ELISA). Data are shown as mean percentage change from baseline (%CfB) at week 8 and week 26 and are represented as mean ± 95% CI **(A)** C-terminal telopeptide I [CTX-I]; **(B)** hyaluronic acid [HA]; **(C)** tissue inhibitor matrix metalloproteinase 1 [TIMP-1]; **(D)** fragment of type III collagen released by MMP [C3M]; **(E)** type II collagen cleavage product [C2C]; **(F)** released N-terminal pro-peptide of type II collagen [Pro-C2]. Outlier analysis was performed on all %CfB data. Data analyzed by a two-way ANOVA with Sidak’s post-test and multiple comparisons comparing PPS and placebo at all timepoints. The n for each group and timepoint corresponds to valid biomarker measurements.

The effect of PPS treatment on serum biomarkers CTX-I, HA, TIMP-1, C3M, C2C, and PRO-C2, was assessed at baseline, weeks 8, and 26 and compared with placebo (Fig 4).

CTX-I, a marker of bone resorption, was reduced compared to baseline (%CfB) in PPS-treated dogs at weeks 8 (−23.8% [−39.42, −8.17]) and 26 (−25.4% [−37.78, −12.99]) and increased in placebo dogs at weeks 8 (1.9% [−15.37, 19.13]) and 26 (8.6% [−9.02, 26.22]). Effect sizes for PPS-treated dogs versus placebo were 1.85, p = 0.06 (−0.717, 50.230), at week 8, and 1.82, p = 0.007 (9.358, 57.430), at week 26 (Fig 4A and S3 Table).

Hyaluronic acid (HA), a marker of systemic inflammation, was marginally increased from baseline at week 8 (3.4% [−18.27, 25.16]) and moderately decreased at week 26 (−9.2% [−37.07, 18.70]) in the PPS-treated group (Fig 4B). Conversely, HA levels reduced at week 8 (−18.1% [−90.21, 54.06]) and increased at week 26 (57.3% [22.06, 92.54]) in placebo. These changes were supported by medium (0.67, p = 0.59 [−75.760, 32.720]) and large (1.57, p = 0.007 [17.050, 115.90]) effect sizes at weeks 8 and 26 respectively.

TIMP-1, an endogenous inhibitor of cartilage degradation, increased following PPS treatment at week 8 (14.1% [−1.81, 30.06]) versus a reduction in placebo (−19.1% [−72.89, 33.29]; effect size 0.96, p = 0.07 [−69.810, 1.960]) (Fig 4C). In contrast, TIMP-1 reduced in both groups at week 26, however the magnitude of change was smaller in the PPS-treated group (−12.1% [−26.99, 2.77]) versus placebo (−35.9% [−67.06, −4.68]), equating to a large effect size of 0.91 (p = 0.24 [−59.630, 12.130]).

Other biomarkers of cartilage degradation (C3M, C2C), and formation (Pro-C2) at weeks 8 and 26 were not substantially altered relative to baseline between placebo and PPS-treated groups (Fig 4D–Fig 4F).

## Discussion

Here, we investigated the DMOAD potential of PPS in naturally-occurring canine OA. DMOADs are defined as drugs that “alter the natural history of disease progression by arresting joint structural change and ameliorating symptoms, either by reducing pain or improving physical function” [32]. Short-term studies have demonstrated that subcutaneous or intramuscular PPS administration in naturally-occurring or surgically-induced canine OA effectively reduces pain and lameness [14,33], improves histological cartilage quality [34], and reduces urinary cartilage degradation products [15].

Although pain is highly subjective, the HCPI indirectly assesses pain via functional outcomes, making it an informative indicator of treatment effects. In this study, a positive treatment effect of PPS on pain (HCPI) versus placebo was sustained until week 26 after MMRM analysis which accounted for differences in baseline pain between groups. Additionally, HCPI is often correlated with gait analysis to determine pain effects on movement. Despite an absence of formal correlation analysis, reduced pain was linked to gait normalization out to week 26 in the PPS-treated group.

Gait analysis is an essential tool in the veterinary clinical workup of dogs with suspected OA, as it enables objective assessment of impaired weight bearing [35]. Smith et al. demonstrated that intramuscular PPS in dogs with spontaneous OA improved lameness and pain (orthopedic score) out to eight weeks (four weeks post final injection) [14]. The current study is the first examining PPS treatment effect duration on gait (lameness) out to 26 weeks (six months) in naturally-occurring canine OA.

A typical gait is often highly symmetrical, with abnormal gait symmetries indicative of orthopedic, muscular, and/or neurologic disorders. Gait analysis provides insights into the compensatory mechanisms contributing to load redistribution, either from medial to lateral (contralateral), or pelvic to thoracic (ipsilateral) in dogs with OA. The most pronounced changes are expected to occur initially in the index limb, then the contralateral limb, the opposing contralateral limb, then the opposing ipsilateral limb [36]. Improvements in TPI% and SI (contralateral and ipsilateral) indicated PPS treatment normalized gait, supported by large to medium effect sizes at weeks 8 and 26.

Quantitative MRI demonstrated potential stabilization of cartilage degeneration in PPS-treated animals with slowed cartilage volume loss at weeks 8 and 26 supported by large to medium effect sizes. Despite regional variations in cartilage volume, changes in total cartilage volume in the PPS-treated group indicated a potentially long-term positive effect on OA disease progression.

Biomarkers of bone and cartilage degradation have been explored in several canine OA studies. Here, PPS treatment modulated several serum biomarkers involved in cartilage degradation processes. PPS treatment reduced levels of the bone degradation marker CTX-I and a marker of systemic inflammation, HA [37] out to week 26, indicating potential extended PPS effects on bone metabolism and inflammation. In contrast, a sustained increase in TIMP-1, the endogenous inhibitor of the cartilage-degrading enzyme ADAMTS-5 was observed in PPS-treated dogs, supporting chondroprotective actions of PPS. These serum biomarker changes could be mediated through PPS inhibition of the transcription factor NF-‍κB via gene expression modulation [8,9].

Interpretation of the results was limited to descriptive statistics due to the small study numbers. Furthermore, as numerous dogs were new to the clinic, detailed clinical histories relating to the origins of OA were not available. A further limitation arose when blinded randomization inadvertently led to the skewing of baseline pain (HCPI), with higher scores in the PPS-treated group versus placebo. Additionally, although HCPI is a validated pain and quality of life assessment tool, it is subject to variability between owners, and is influenced by environment, dog breed, age, gender, and origin of pain. Another study limitation related to rescue medication use in the placebo group. The rescue medication, grapiprant, targets the prostaglandin EP4 receptor without suppressing prostaglandin production. As such, grapiprant should have minimal effects on PPS mechanisms of action. Based on grapiprant washout periods, it is possible that any HCPI and gait improvement in placebo dogs receiving rescue medication may have been due to grapiprant. Lastly, despite collecting synovial fluid samples from some dogs, due to limited sample volume, high sample viscosity and assay reactivity to canine antigens, assay validation in this matrix was prohibitive and consequently samples were not further analyzed or reported in this study.

Despite small study numbers in this pilot study, we observed long-lasting PPS treatment effects out to 26 weeks, supported by medium to large effect sizes at the functional, structural, and molecular levels. More specifically, this study established that PPS treatment improved canine gait by normalizing balance between limbs (SI), stabilizing cartilage volume in the stifle joint, reducing levels of serum biomarkers of cartilage degradation (CTX-I, HA), and increasing levels of an inhibitor of matrix-degrading enzymes (TIMP-1). These results could explain previous findings when treating canine OA with PPS [13–16]. This canine model is potentially advantageous in rapidly evaluating PPS DMOAD effects that would otherwise require a longer assessment period for joint structural changes in humans. These results could inform future study designs and effect size calculations for larger clinical trials in dogs and humans.

## Supporting information

S1 Table Study schedule. (DOCX)

S2 Table Clinical biochemistry and hematology analysis. (DOCX)

S3 Table Effect size (ES) analysis of percentage change from baseline (%CfB) datasets. (DOCX)
