# Supplementary material for: Effects of pentosan polysulfate sodium on joint structure and function out to six months in naturally-occurring canine osteoarthritis
Source: PLoS One. 2026 Feb 10;21(2):e0342409. doi: 10.1371/journal.pone.0342409 (PMC12890089; doi:10.1371/journal.pone.0342409)
Supplement: S1 Table — (DOCX) [file pone.0342409.s001.docx]

## S1 Table: Study Schedule

| **Study Week** | **Baseline (0)** | **1** | **2** | **3** | **4** | **5** | **6** | **8** | **26** |
| --- | --- | --- | --- | --- | --- | --- | --- | --- | --- |
| Pre-screening | X |  |  |  |  |  |  |  |  |
| Gait analysis | X |  |  | X |  |  | X | X | X |
| Helsinki Chronic Pain Index (HCPI) Survey | X |  |  | X |  |  | X | X | X |
| X-ray | X |  |  |  |  |  |  |  | X |
| Blood Collection | X | X |  |  |  |  |  | X | X |
| Randomization |  | X |  |  |  |  |  |  |  |
| Subcutaneous injections |  | X | X | X | X | X | X |  |  |
| Synovial fluid collection |  | X |  |  |  |  |  | X | X |
| Magnetic resonance imaging (MRI) |  | X |  |  |  |  |  | X | X |
| Urine collection |  | X |  |  |  |  |  | X | X |

Companion dogs presenting at U-Vet Animal Hospital for lameness assessment were screened at week 0 (baseline). The maximum time between week 0 (screening) and dose 1 was two weeks. If eligible, dogs underwent study group randomization in a 2:1 ratio (PPS to placebo), MRI, urine, blood (serum), and synovial fluid collection. Subcutaneous 3 mg/kg PPS injections (1.7 mg/kg human equivalent dose) were administered weekly for 6 weeks. HCPI and gait analyses were performed at weeks 3 and 6. At week 8 (2 weeks after final injection), dogs underwent MRI, blood (serum) and urine collection, HCPI, and gait analysis. At the final week 26 (6-month) timepoint, dogs underwent all analyses, including X-ray. Abbreviations: HCPI, Helsinki Chronic Pain Index; MRI, magnetic resonance imaging; PPS, pentosan polysulfate sodium.
