# Supplementary material for: Effects of pentosan polysulfate sodium on joint structure and function out to six months in naturally-occurring canine osteoarthritis
Source: PLoS One. 2026 Feb 10;21(2):e0342409. doi: 10.1371/journal.pone.0342409 (PMC12890089; doi:10.1371/journal.pone.0342409)
Supplement: S2 Table — (DOCX) [file pone.0342409.s002.docx]

## S2 Table: Clinical Biochemistry and Hematology Analysis

| **Clinical Biochemistry and Hematology** | | | | **PPS** | | **Placebo** | |
| --- | --- | --- | --- | --- | --- | --- | --- |
| **Parameter** | **Units** | **Lower Limit** | **Upper Limit** | **Baseline** | **Week 26** | **Baseline** | **Week 26** |
| Red cell count | × 10^12^/L | *5.7* | *8.5* | 7.0 | 6.8 | 7.3 | 6.9 |
| Haemoglobin | g/L | *141* | *201* | 164.1 | 159.5 | 172.0 | 159.5 |
| Haematocrit | L/L |  |  | 0.4 | 0.4 | 0.5 | 0.4 |
| MCV (HCT/RCC) | fL | *64* | *76* | 64.5 | 65.1 | 64.2 | 63.2 |
| MCH (Hb/RCC) | pg | *21* | *26* | 23.6 | 23.4 | 23.4 | 24.0 |
| MCHC (Hb/HCT) | g/L | *330* | *360* | 366.4 | 362.0 | 365.4 | 363.3 |
| RDW-SD | fL |  |  | 32.6 | 32.8 | 31.6 | 31.2 |
| Platelets | × 10^9^/L | *186* | *545* | 323.5 | 355.7 | 260.4 | 312.2 |
| NRBC% | % | *0* | *1* | 0.2 | 0.1 | 0.1 | 0.1 |
| White cell count | × 10^9^/L | *5.7* | *14.2* | 8.3 | 8.2 | 9.6 | 8.3 |
| Neutrophils | × 10^9^/L | *2.7* | *9.4* | 5.9 | 6.0 | 7.2 | 6.0 |
| Lymphocytes | × 10^9^/L | *0.9* | *4.7* | 1.6 | 1.5 | 1.5 | 1.5 |
| Monocytes | × 10^9^/L | *0.1* | *1.3* | 0.4 | 0.4 | 0.5 | 0.4 |
| Eosinophils | × 10^9^/L | *0.1* | *2.1* | 0.3 | 0.5 | 0.4 | 0.3 |
| Reticulocytes | × 10^9^/L | *11* | *92* | 35.2 | 44.0 | 63.4 | 50.0 |
| Ret-H*e* | pg | *29.8* | *37.7* | 24.1 | 24.1 | 24.3 | 23.6 |
| PT | seconds | *7* | *9.8* | 8.6 | 8.8 | 8.7 | 9.2 |
| aPTT | seconds | *10.8* | *14.7* | 14.6 | 13.3 | 14.2 | 13.0 |
| Sodium | mmol/L | *140* | *154* | 150.8 | 147.5 | 145.0 | 149.6 |
| Potassium | mmol/L | *3.8* | *5.4* | 4.7 | 4.7 | 4.3 | 4.4 |
| Chloride | mmol/L | *104* | *119* | 116.1 | 116.9 | 112.4 | 119.2 |
| Calcium | mmol/L | *2.5* | *3* | 2.6 | 2.7 | 2.5 | 2.6 |
| Phosphate | mmol/L | *0.8* | *1.9* | 1.2 | 1.2 | 1.0 | 1.2 |
| Urea | mmol/L | *3.5* | *9* | 5.9 | 5.8 | 5.7 | 6.0 |
| Creatinine | µmol/L | *20* | *150* | 87.7 | 85.2 | 102.6 | 91.5 |
| Glucose | mmol/L | *3.3* | *7.3* | 6.1 | 6.2 | 6.0 | 6.7 |
| Cholesterol | mmol/L | *3.6* | *10.2* | 6.8 | 6.8 | 6.1 | 6.8 |
| Bilirubin Total | µmol/L | *0* | *4* | 0.6 | 0.8 | 1.2 | 0.6 |
| ALT | U/L | *17* | *95* | 40.2 | 89.2 | 69.2 | 63.5 |
| ALP | U/L | *22* | *143* | 75.5 | 97.3 | 51.0 | 111.4 |
| GGT | U/L | *0* | *7* | 1.7 | 2.2 | 1.6 | 1.4 |
| Amylase | U/L | *322* | *1310* | 846.1 | 930.6 | 628.0 | 714.8 |
| Lipase | U/L | *15* | *228* | 87.7 | 67.7 | 38.4 | 36.6 |
| CK | U/L | *64* | *314* | 111.7 | 107.5 | 126.0 | 105.6 |
| Total protein | g/L | *59* | *78* | 64.8 | 67.5 | 62.0 | 65.4 |
| Albumin | g/L | *31* | *37* | 40.1 | 39.8 | 38.6 | 38.0 |
| Globulin | g/L | *23* | *34* | 25.4 | 28.4 | 23.4 | 27.4 |
| AST | U/L | *18* | *56* | 27.5 | 29.5 | 37.0 | 32.5 |

Abbreviations: L, liter; g, gram; MCV, mean corpuscular volume; HCT, hematocrit; RCC, red cell count; fL, femtolitre; MCH, mean corpuscular hemoglobin; Hb, hemoglobin; pg, picogram; MCHC, mean corpuscular hemoglobin concentration; RDW-SD, red cell distribution width - standard deviation; NRBC%, nucleated red blood cell percentage; Ret-He, reticulocyte hemoglobin content; PT, prothrombin time; aPTT, activated partial thromboplastin time; mmol/L, millimolar per liter; µmol/L, micromolar per liter; ALT, alanine aminotransferase; ALP, aspartate aminotransferase; GGT, gamma-glutamyl transferase; U/L, units per liter; CK, creatinine kinase; AST, aspartate aminotransferase; PPS, pentosan polysulfate sodium.
