# Supplementary material for: Effects of pentosan polysulfate sodium on joint structure and function out to six months in naturally-occurring canine osteoarthritis
Source: PLoS One. 2026 Feb 10;21(2):e0342409. doi: 10.1371/journal.pone.0342409 (PMC12890089; doi:10.1371/journal.pone.0342409)
Supplement: S3 Table — (DOCX) [file pone.0342409.s003.docx]

## S3 Table: Effect Size (ES) Analysis of Percentage Change from Baseline (%CfB) Datasets.

| **Readout** | **Week 8 ES** | **Description** | **Week 26 ES** | **Description** |
| --- | --- | --- | --- | --- |
| Total Pressure Index (TPI%) | 0.83 | Large | 0.68 | Medium |
| Cartilage Vol (Total) | 1.06 | Large | 0.73 | Medium |
| Cartilage Vol (Patella) | 0.32 | Small | 0.18 | Small |
| Cartilage Vol (Tibial Plateau) | 0.047 | NA | 0.40 | Medium |
| Cartilage Vol (Femoral Condyle) | 1.19 | Large | 0.11 | Small |
| CTX-I | 1.15 | Large | 1.82 | Large |
| C2C | 0.72 | Medium | 0.43 | Small |
| C3M | 0.35 | Small | 0.26 | Small |
| HA | 0.58 | Medium | 1.57 | Large |
| Pro-C2 | 0.26 | Small | 0.36 | Small |
| TIMP-1 | 0.96 | Large | 0.91 | Large |

Effect sizes (ES) are independent of the sample size and are categorized as small (0.20–0.49), medium (0.50–0.79), or large (>0.80). Abbreviations: ES, effect size; %CfB, percentage change from baseline; HCPI, Helsinki chronic pain index; TPI%, total pressure index percentage; Vol, volume; CTX-I, c-terminal telopeptide I; C2C, cartilage collagen neoepitope; C3M, collagen type III degradation product; HA, hyaluronic acid; Pro-C2, N-terminal propeptide of type IIB collagen; TIMP-1, tissue inhibitor of metalloproteinase 1, NA: not applicable.
